# Feasibility, Safety, and Performance of Full-Head Subscalp EEG Using Minimally Invasive Electrode Implantation

**DOI:** 10.1212/WNL.0000000000209428

**Published:** 2024-06-06

**Authors:** Ellen van Maren, Sigurd L. Alnes, Janir Ramos da Cruz, Aleksander Sobolewski, Cecilia Friedrichs-Maeder, Katharina Wohler, Sabry L. Barlatey, Sandy Feruglio, Markus Fuchs, Ioannis Vlachos, Jonas Zimmermann, Tiago Bertolote, Werner J. Z'Graggen, Athina Tzovara, John Donoghue, George Kouvas, Kaspar Schindler, Claudio Pollo, Maxime O. Baud

**Affiliations:** From the NeuroTec (E.v.M., S.L.A., C.F.-M., K.W., S.F., M.F., A.T., K.S., M.O.B.), Center for Sleep-Wake-Epilepsy, Center for Experimental Neurology, Department of Neurology, Inselspital Bern, University Hospital, and Institute of Computer Science (S.L.A., A.T.), University of Bern; Wyss Center for Bio and Neuroengineering (J.R.d.C., A.S., I.V., J.Z., T.B., G.K.), Geneva; Department of Neurosurgery (S.L.B., W.J.Z.G., C.P.), Inselspital Bern, University Hospital, University of Bern, Switzerland; and Department of Neuroscience (J.D.), Brown University, Providence, RI.

## Abstract

**Background and Objectives:**

Current practice in clinical neurophysiology is limited to short recordings with conventional EEG (days) that fail to capture a range of brain (dys)functions at longer timescales (months). The future ability to optimally manage chronic brain disorders, such as epilepsy, hinges upon finding methods to monitor electrical brain activity in daily life. We developed a device for full-head subscalp EEG (Epios) and tested here the feasibility to safely insert the electrode leads beneath the scalp by a minimally invasive technique (primary outcome). As secondary outcome, we verified the noninferiority of subscalp EEG in measuring physiologic brain oscillations and pathologic discharges compared with scalp EEG, the established standard of care.

**Methods:**

Eight participants with pharmacoresistant epilepsy undergoing intracranial EEG received in the same surgery subscalp electrodes tunneled between the scalp and the skull with custom-made tools. Postoperative safety was monitored on an inpatient ward for up to 9 days. Sleep-wake, ictal, and interictal EEG signals from subscalp, scalp, and intracranial electrodes were compared quantitatively using windowed multitaper transforms and spectral coherence. Noninferiority was tested for pairs of neighboring subscalp and scalp electrodes with a Bland-Altman analysis for measurement bias and calculation of the interclass correlation coefficient (ICC).

**Results:**

As primary outcome, up to 28 subscalp electrodes could be safely placed over the entire head through 1-cm scalp incisions in a ∼1-hour procedure. Five of 10 observed perioperative adverse events were linked to the investigational procedure, but none were serious, and all resolved. As a secondary outcome, subscalp electrodes advantageously recorded EEG percutaneously without requiring any maintenance and were noninferior to scalp electrodes for measuring (1) variably strong, stage-specific brain oscillations (alpha in wake, delta, sigma, and beta in sleep) and (2) interictal spikes peak-potentials and ictal signals coherent with seizure propagation in different brain regions (ICC >0.8 and absence of bias).

**Discussion:**

Recording full-head subscalp EEG for localization and monitoring purposes is feasible up to 9 days in humans using minimally invasive techniques and noninferior to the current standard of care. A longer prospective ambulatory study of the full system will be necessary to establish the safety and utility of this innovative approach.

**Trial Registration Information:**

clinicaltrials.gov/study/NCT04796597.

## Introduction

Since Berger's first application of electrodes to the human scalp and his discovery of the alpha rhythm in 1929, recording capabilities of the EEG have steadily increased in terms of spatial coverage (increasing number of electrodes), duration (over days), and digitization. Early on, scalp EEG had become the state-of-the-art method for diagnosing transient neurologic dysfunctions, such as those seen in epilepsy or sleep disorders. At present, the Internet of Things affords increasing data management capabilities, and there emerges the potential to elevate EEG from a diagnostic test to a chronic monitoring system. Such transformation could mirror the significant paradigm shift witnessed in cardiology with the advent of implantable devices and redefine contemporary practice in clinical neurophysiology. However, ultra-long-term EEG recordings over months have been limited by the instability of scalp EEG and the invasiveness of intracranial systems.^1^

As a method, conventional scalp EEG excels at the millisecond resolution of evanescent waveforms that appear at specific scalp locations and signal the physiologic alternance of vigilance states or pathologic processes. Specifically, conventional EEG has significant clinical advantages in epileptology: (1) It is widely available; (2) it can faithfully record seizures that a patient may or may not notice or remember^2^; (3) it helps understand the interplay between a patient's sleep-wake cycle and seizures^3^; and (4) it can reveal the lobar to sublobar localization of focal seizures, which varies across or even within patients.^4,5^ However, conventional scalp EEG also has serious limitations: Timely recording is essential to capture fleeting signatures of dysfunction, but wearing and maintaining an EEG at all times on the scalp is impractical and stigmatizing.^6^ To ensure sufficient quality of the recordings, electrodes must be patiently stuck between hairs and verified on a daily basis.^7^ To achieve this, hospitals have developed costly EEG monitoring units in which patients' mobility is restricted over days to weeks by cabling to bulky bedside instrumentation. Thus, despite the strengths of conventional EEG (temporal resolution and spatial coverage), the limited duration of recording hampers its usefulness in clinical practice.^8^

In the past decade, a number of efforts attempted to address the unmet need for unobtrusive monitoring of epileptic brain activity outside the clinic without a fully satisfactory solution to date. Current intracranial systems approved as the last resort for palliative neurostimulation in pharmacoresistant epilepsy offer a very restricted EEG recording capability of a few minutes per day.^9,10^ A less invasive, but chronic approach, called “subscalp EEG,” wherein electrodes are inserted between the scalp and the cranium, has attracted attention as a more promising solution for ambulatory EEG monitoring.^11,12^ One such system was CE-marked in 2019 (UNEEG Medical's 24/7 EEG SubQ System), and another is in clinical trials (Epiminder's Minder System). Already, preliminary data have shown that these systems can help count seizures and capture cycles of epileptic brain activity.^13-15^ However, their limited spatial coverage (2 bipolar electrodes^11^) does not enable the localization of physiologic oscillations or pathologic discharges, an issue particularly important in epilepsy, for which guidelines recommend using a minimum of 16–25 electrodes.^7,16^

Starting in 2017, the Wyss Center for Bio and Neuroengineering in collaboration with the Inselspital conceived a system called Epios to combine full-head coverage with ultra-long-term recording over many months. The patented innovation involves an implantable trident-shaped electrode lead that is inserted between the scalp and the cranium (hereafter “trident”). As a primary outcome of the present short-term, hospital-based clinical trial, we tested the feasibility of safely inserting and maintaining up to 4 Epios tridents (28 electrode contacts) to sample subscalp EEG from the entire head. As a secondary outcome, we assessed the noninferiority of subscalp vs scalp electrodes in recording qualitative EEG signals percutaneously and their ability to localize physiologic and pathologic signals across the sleep-wake cycle.

## Methods

### Standard Protocol Approvals, Registrations, and Patient Consents

We performed a single-arm, prospective, early-feasibility, clinical investigation of a medical device at a single center (Inselspital, Bern University Hospital, Bern, Switzerland) sponsored by the Wyss Center for Bio and Neuroengineering, a nonprofit organization. The trial entitled “Early feasibility study on Epios leads” was registered on ClinicalTrials.gov on March 15, 2021 (NCT04796597), after obtaining authorization from Swissmedic (#10000731, EUDAMED CIV-20-07-034045) and the ethics commission of Canton Bern (#2019-00723).

Participants were chronologically recruited from our neurosurgical clinic (Neurozentrum, Inselspital, Bern, Switzerland) between March 2021 and July 2023 among adults (18 years and older) undergoing brain surgery for clinical reasons. Exclusion criteria for trial participation were severe neuropsychiatric disorder, severe cognitive problems, severe chronic headache disorder, and the presence of hardware on the head, as well as severe medical conditions contraindicating cranial surgery. Participants diagnosed with pharmacoresistant epilepsy were video-EEG monitored 24/7 in an intermediate care unit for presurgical seizure localization using intracranial EEG per clinical routine. After a minimal reflection time of 3 days upon receiving the written and oral study information, all participants signed informed consent to have additional subscalp electrodes implanted for research purposes and without any clinical benefit.

### Medical Device

**Figure 1:**
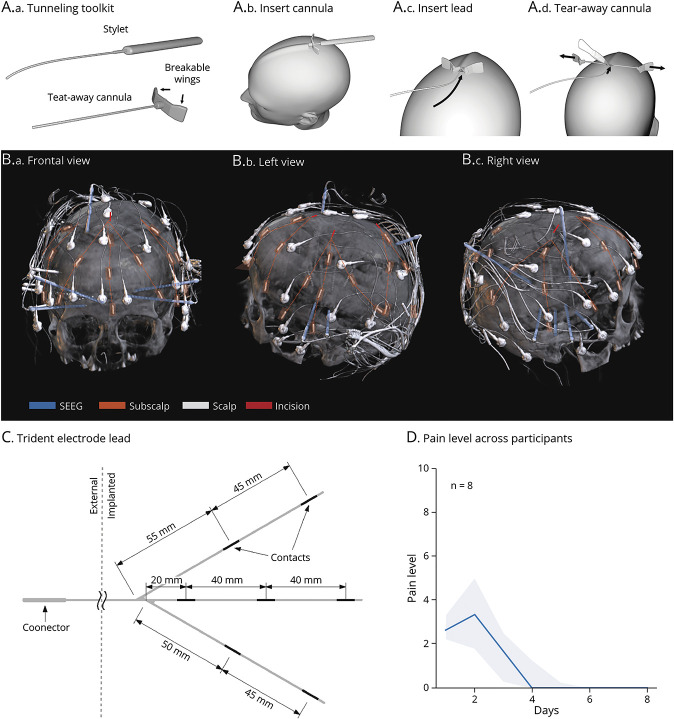
Surgical Procedure and Postoperative Outcomes (A) Tunneling toolkit (A1) containing stylets and tearable cannulas specifically designed and validated for the subscalp insertion of the Epios “trident” lead. In order from left to right, the branches of the trident are inserted one by one by (A2) pushing the cannula under the scalp, using the rigid stylet, (A3) retracting the stylet and inserting 1 branch of the trident into the cannula and (A4) tearing away the cannula to leave the branch in place (held with a tweezer, A4). (B) Example in 1 participant of the 3-dimensional postoperative CT scan showing the skull-penetrating depth electrodes (blue, clinical procedure), the full-head coverage with 4 subscalp tridents (orange, investigational procedure), and the conventional scalp EEG electrodes (white, comparator recording). Orange dotted lines highlight the trident electrode lead with 7 contacts. The 4 red lines illustrate the ∼10-mm incisions used for the insertion of each trident. All electrode leads were connected percutaneously to the same EEG amplifier (wires at the back of the head, see also eFigure 2). (C) Trident electrode lead showing the 3 branches with 7 contacts in total with the distance between contacts. (D) Median (IQR) pain level (NRS 0–10) over postoperative days across participants (n = 8). Note that all participants contributed data until day 5, thereafter only a portion. IQR = interquartile range; NRS = Numerical Rating Scale.

Between 2017 and 2021, the trident lead and its implantation procedure was first designed, iteratively developed, and tested in 6 anatomical studies with human cadavers at the institute for anatomy of the Universities of Bern and Lausanne. In a preliminary clinical test, the device performance and its implantation were validated intraoperatively on 2 patients undergoing brain surgery (step 1, eFigure 1). This test allowed for the selection of a final lead design for the clinical trial (step 2, main results). The tested medical device (Figure 1) consists of the Epios subscalp lead (manufactured by Cortec GmBH, Freiburg, Germany) and custom-made Epios insertion tools: tearable soft cannulas and rigid stylets (manufactured by Medlight SA, Ecublens, Switzerland). One trident lead entails 1 trunk and 3 branches (1.3 mm diameter), which join into a trifurcation. It is made of medical-grade silicon with 7 platinum-iridium contacts in total. The proximal end of the trunk has a connector to our standard clinical EEG monitoring system for percutaneous recordings. The Epios lead is classified as a medical device, class IIb, and was verified according to all relevant standards. In April 2023, the complete patented Epios system (Patent Cooperation Treaty #62665486) was granted with Breakthrough Device Designation by the US Food and Drug Administration for patients with drug-resistant epilepsy.

### Implantation Procedure

In a single surgery under general anesthesia, participants first underwent the clinically indicated procedure (n = 1 subdural EEG grid and n = 7 intraparenchymal stereo-EEG, Table 1) and then received 1–4 investigational subscalp electrode lead(s). Using the custom-made tunnelization tools, each trident was inserted beneath the scalp through a ≤1 cm incision made in 1 of 4 locations: left or right temporal, frontal, or parietal. From this single-entry point, 3 cannulas were pushed over 10–12 cm under the scalp in 3 different directions (∼30° angle between trajectories), using the guiding and dissecting stylet. After removing the stylet, 3 trident branches were pushed into the tearable cannula and kept in place while the cannula was torn away. The trunk of the trident was also tunneled over 3–5 cm to exit through a 1-mm puncture hole in the opposite direction for percutaneous connection (Figure 1A). Immediately after implantation, a 10-20 scalp EEG was mounted (25 electrodes), and a brain CT was obtained and aligned to the participant's presurgical MRI, as per clinical routine (Supplement). All distances were measured on the 3-dimensional reconstruction of the brain and implanted electrodes.

**Table:** Participant Characteristics and Adverse Events

ID	Age, y/sex	Epilepsy	Etiology	Lat	No. of days	No. of contacts	F	TL	TR	P	ADE	No. of seizures recorded
P1	25/F	nTLE speech cortex	Post-traumatic	L	7	7	—	✓	—	—	None	7
P2	20/F	mTLE	Hippocampal sclerosis	R	5	14	—	✓	✓	—	Painful incision	13
P3	45/F	mTLE	DNET	L	5	14	✓	✓	—	—	None	8
P4	32/M	Perisylvian cortex	Perinatal ischemia	L	5	14	✓	—	—	✓	None	1
P5	55/M	mTLE	Hippocampal sclerosis	L	7	28	✓	✓	✓	✓	Scalp edema	11
P6	63/F	nTLE	Unknown, nonlesional	R	7	28	✓	✓	✓	✓	Scalp perforation	2
P7	26/M	mTLE	Unknown, nonlesional	R	9	28	✓	✓	✓	✓	Subscalp hematoma	3
P8	56/F	mTLE	Unknown, nonlesional	R	5	28	✓	✓	✓	✓	Scalp perforation	6

Abbreviations: ADE = nonserious adverse device effect possibly or definitely related to the investigational procedure (details in eTable 2); DNET = dysembryoplastic neuroepithelial tumor; F = female; M = male; mTLE = mesiotemporal lobe epilepsy; nTLE = neocortical temporal lobe epilepsy. Subscalp trident implantation: F = frontal; TL = temporal left; TR = temporal right; P = parietal.

Note that all participants were also implanted with an intracranial EEG and had a 25 electrode scalp EEG for clinical reasons. P1 received a subdural grid, all others received intraparenchymal stereo-EEG leads.

### Safety Outcomes

All participants contributed to the primary analysis of feasibility and safety. The implantation procedure was timed, and any immediate adverse event was documented. Postoperative head pain level was serially assessed, first hourly then less frequently, as per clinical routine, using a numerical scale from 0 (“no pain at all”) to 10 (“the worst pain imaginable”). The participant's head was regularly examined every 1–2 days for postoperative scalp edema, hematoma, puncture hole, or pain upon palpation of the subscalp leads.

### Device Performance and Signal Recording

All EEG analyses were performed on all available subscalp EEG channels and compared visually, spectrally, and topographically with scalp EEG (comparator, established standard of care) or intracranial EEG (ground truth for epileptiform discharges) amplified and digitized by a single amplifier and processed in the same manner (Supplement). The secondary performance outcome of the study was the ability of the medical device to provide EEG signal that is noninferior to that recorded by scalp EEG. We compared paired measurements of voltage and oscillatory power or coherence (see below) from each subscalp electrode against its closest scalp electrode neighbor.

#### Visual Sleep-Wake Scoring

To test the ability to assess the sleep-wake architecture from subscalp EEG recordings as compared with scalp EEG, 3 trained unblinded scorers (2 certified clinical neurophysiologists, C.F.-M and K.W., and 1 trained technician/PhD student E.v.M.) double-scored 1 full 24-hour recording period from each participant: once with subscalp and once with scalp EEG according to the guidelines of the American Academy of Sleep Medicine Manual for the Scoring of Sleep and Associated Events.^17^ Cohen's κ agreement was calculated across scorers and recording modality to assess inter and intrarater agreement, respectively, and differences were tested using a Wilcoxon sign test. The agreement coefficient ranges from 0 (chance agreement) to 1 (identical scoring), and a prespecified coefficient >0.60 represents a substantial agreement,^18^ which we used as a margin limit for noninferiority.

#### Evoked Potentials

To assess the ability of subscalp EEG to measure evoked potentials, 2 and 3 participants received visual (flickering checkerboard) and/or auditory (pure tones) stimuli, respectively (Supplement).

#### Spectral Analysis

We obtained estimates of the power spectral density by applying the multitaper spectral method over 5-second sliding windows with a 50% overlap^19^ using custom Python scripts and the MNE package version 0.24.^20^ To measure the similarities between 2 signals in time and frequency, we calculated the coherence between their corresponding multitaper-derived spectra (Supplement).

#### Topographical Analysis

To visualize the distribution of brain activity over the entire head, we created topographic maps in the subset of n = 4 participants who received 4 tridents using the MNE Python package. For all such “topoplots” generated from oscillatory power or peak voltage amplitude, we calculated the spatial correlation coefficient to quantify the topographical relationship between subscalp and scalp EEG and tested its significance against a null distribution of correlation values. Prespecified Pearson's ρ >0.5 and >0.7 were considered moderate and strong, respectively.

#### Ictal Discharges

Qualitatively, 2 unblinded expert reviewers (C.F.-M and K.W.) visually scored whether seizures recognized in the clinical workup were recognizable in the subscalp and scalp EEG recordings as an ictal discharge evolving in space, amplitude, and frequency typically over 1–2 minutes. Quantitatively, comparing the signal quality during seizures requires a refined spatiotemporal approach because seizures evolve in space, time, and frequency in a patient-specific manner. To quantify the similarity between subscalp and scalp recordings of seizures, we compared them directly and also with the ground truth intracranial EEG in the seizure onset zone (SOZ) and propagation zone (PZ). To do so, we first identified the subscalp contact closest to the SOZ. Second, the scalp and intracranial contacts closest to this selected subscalp contact were identified. These 3 contacts recording 3 modalities (subscalp, scalp, and intracranial) were expected to be at a distance from the SOZ, making them more comparable in terms of spectral coherence (0.5–30 Hz).

#### Interictal Discharges

To identify 30 interictal spikes, an expert reviewer (K.W.) visually inspected the intracranial EEG data and marked the most prominent and frequent spike(s) with an electrical field extending to the cortex on the convexity of the brain. To evaluate the electrical dipole at its maximal intensity, the peak voltage amplitude of the peak-aligned average spike was analyzed and compared.

### Statistics

The primary study outcome was reported as numerical values without statistical testing. As secondary outcome, noninferiority of subscalp EEG was accepted for measurement values at least not inferior to −20% to established values for scalp EEG, that is, −1 μV for voltage, −0.5 dB for oscillatory peak power, and −0.03 point for oscillatory coherence. Following the Bland-Altmann methodology for measurement agreement^21^ across participants and pairs of electrodes, we tested for a (1) strong interclass correlation coefficient^22^ (ICC >0.75, python package pingouin^23^) and (2) a lack of bias (mean difference in measured values) inferior to the above preset criterion (Supplement). As other outcomes of interest, we compared the obtained spatial correlation of topoplots derived from subscalp and scalp EEG with that of surrogate data, in which the data were shuffled 100 times to generate a permutation distribution (Supplement). The study protocol and statistical analysis plan are available in eSAP 1.

### Data Availability

Anonymized data not published within this article may be made available upon reasonable request from a qualified investigator to Inselspital and Wyss Center for Bio and Neuroengineering.

## Results

Between March 2021 and July 2023, 8 of 14 screened adults with focal epilepsy of unclear localization (3 male participants, median [range] age: 38 years [20–63], Table 1) agreed to receive 1–4 investigational electrode leads (so-called tridents), in addition to the intracranial electrodes necessary for presurgical localization of their seizures. In total, 23 subscalp tridents (161 contacts) were implanted for a median duration of 6 days (5–9). Participants received a variable number of intracranial electrodes according to their clinical needs, and all had a conventional scalp EEG (25 contacts) for comparison. The median number of seizures recorded per patient was 7.5 (1–13) from various neocortical or mesiotemporal sources (Table 1). All participants' data contributed to the safety, performance, and spectral analyses (P1–8). In addition, topoplots of oscillations or potentials of interest were derived from the 4 participants who received 4 tridents (P5–8).

### Safety Outcomes

The surgical tools and the insertion procedure (Figure 1A) were deemed fit for the intended use by the chief neurosurgeon involved in the trial (C.P.). Across participants, the median (range) insertion time for 1 subscalp trident, involving the tunnelization of 3 branches, was 12 minutes (8–21). The median (range) incision length for the insertion of the tridents was 10 mm (9–11) (eTable 1, eFigure 2). Postoperative CT and preoperative MRI alignment revealed the expected positioning of the electrodes (Figure 1, B and C). We did not observe any early explantation nor device-related serious adverse events. We did observe 5 minor adverse events possibly or definitely related to the investigational implantation procedure, all handled conservatively without further complication: 1 painful incision, 2 scalp hematoma or edema, and 2 scalp perforations (Table 1, eTable 2). In addition, we observed 2 serious adverse events and 3 adverse events unrelated to the investigational device or procedure (eTable 2). Serial postoperative pain assessment revealed a median (interquartile range [IQR]) day-averaged head pain level of 2.7 (2.2–3.3) on the first day, which was resolved by day 4 (0.0 [0.0–1.3]) across all participants (Figure 1D).

### Performance of the Medical Device

The investigational device recorded high-quality subscalp EEG signal 91.0 ± 0.04% of the time across participants and channels (eTable 3). In some cases, we encountered device deficiencies in the form of lost signals from the subscalp recording electrodes. This was due to wire breakages, at/near the extracorporeal connector to downstream equipment as demonstrated by postexplantation micro-CT inspection (eFigure 3). By contrast, the implanted portion of the trident (distal from the trifurcation) and connections to their electrode contacts had remained intact upon inspection after explantation, suggesting their robustness to handling during surgery.

### Wake EEG Recordings

**Figure 2:**
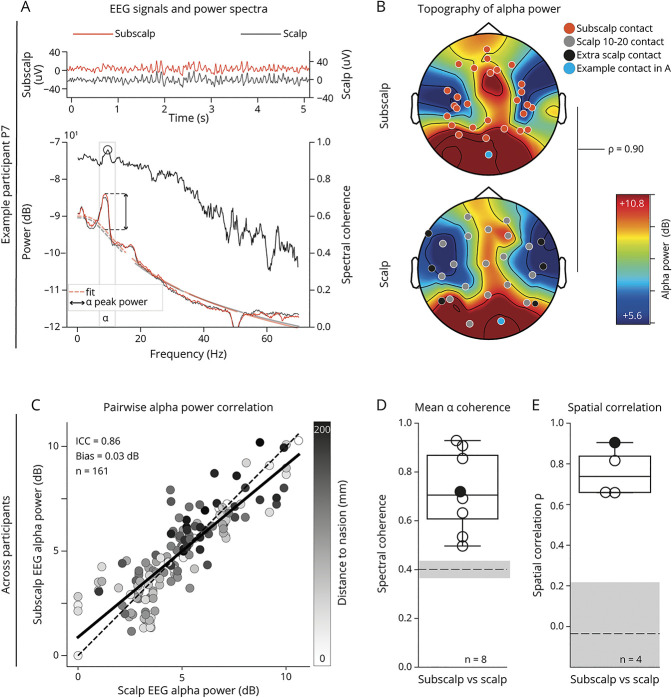
Subscalp and Scalp EEG Recordings During Quiet Wake (A) Example of subscalp compared with scalp EEG signals (5 seconds excerpt from total 120 seconds) in 1 participant (P7) during quiet wake with eyes closed (subscalp: EP1, orange, left y-axis; scalp: O2, gray, right y-axis). Location of electrodes visible in blue on the corresponding topoplots in B. Corresponding multitaper power spectral density on a logarithmic scale (full lines, left y-axis) against frequencies 0–70 Hz from 120-second signals with the corresponding 1/f fits as light dashed lines. The alpha peak power is calculated as the maximal departure from the 1/f fit (double arrow, see Methods). The spectral coherence between the 2 signals is also shown against the right y-axis (black line). The black empty dot indicates peak coherence in the alpha frequency range (gray shadow) reported in D. (B) Corresponding topographic distribution (topoplot) of alpha peak power from 120-second subscalp (top) and scalp EEG (bottom). Black dots indicate inferior temporal scalp contacts added to the conventional 10-20 EEG system and included in the topoplot calculation. The spatial correlation between the 2 topoplots is indicated to the right. (C) Scatterplot of alpha peak power measured in decibels by subscalp vs scalp EEG during quite wake across participants (n = 8). Dots correspond to pairs of neighboring subscalp and scalp contacts (n = 161), and gray shading highlights the expected anteroposterior gradient in alpha power. The linear fit (black line) is visually compared with the identity line (dotted diagonal). The corresponding ICC is shown, and Bland-Altman bias calculations are in eFigure 4. (D) Boxplot showing the median and IQR peak alpha coherence calculated across all neighboring subscalp-scalp electrode pairs for each participant (dots, n = 8). The dashed horizontal line (median) and gray shading (95th percentile) indicate the CI for surrogate data. (E) Boxplot (median an IQR) showing the spatial correlation between subscalp and scalp EEG in each participant implanted with 4 tridents (n = 4). Black dots in D and E correspond to the example participants in plots A and B. ICC = interclass correlation coefficient; IQR = interquartile range.

Visually, examples of wake signals obtained from subscalp electrodes were nearly identical to those obtained from the comparator scalp electrodes (Figure 2A) with similar topographic distribution (Figure 2B). Across participants (n = 8), a multitaper spectral analysis on 120 seconds of EEG signals recorded with eyes closed showed that the investigational subscalp contacts measured posterior-dominant peak alpha power that was not different from neighboring conventional scalp electrodes (Figure 2C). Indeed, the Bland-Altman analysis revealed a mean difference of 0.03 dB (95% CI [−0.15 to 0.21]) above the preset limit of noninferiority of −0.5 dB as well as a strong correlation between subscalp and scalp measurements (ICC [95% CI] = 0.86 [0.82–0.90], Figure 2C, eFigure 4, eTable 4). This result demonstrates noninferiority of subscalp vs scalp EEG in recording quiet wake alpha oscillations. Nearest-neighbor pairs of subscalp and scalp electrodes also gave coherent signals in the alpha range (median [IQR] C = 0.71 [0.61–0.86], Figure 2D). The median (IQR) spatial correlation between topoplots was moderate to strong between the 2 recording modalities with ρ = 0.74 (0.66–0.83) (Figure 2E, n = 4). The spatial correlation is different from the pairwise correlation (ICC above) because it accounts for the spatial location of all electrodes in place to estimate topographic fields. In addition, EEG signal quality during active wakefulness was tested in several situations, including walking, talking, moving the tongue, brushing the teeth, and speaking on the cell phone. These did not lead to any artifacts in the subscalp or scalp recordings. Conversely, chewing, reading, or moving the eyes led to the expected muscle and eye movement artifacts, respectively (eFigure 5).

### Evoked Potentials

**Figure 3:**
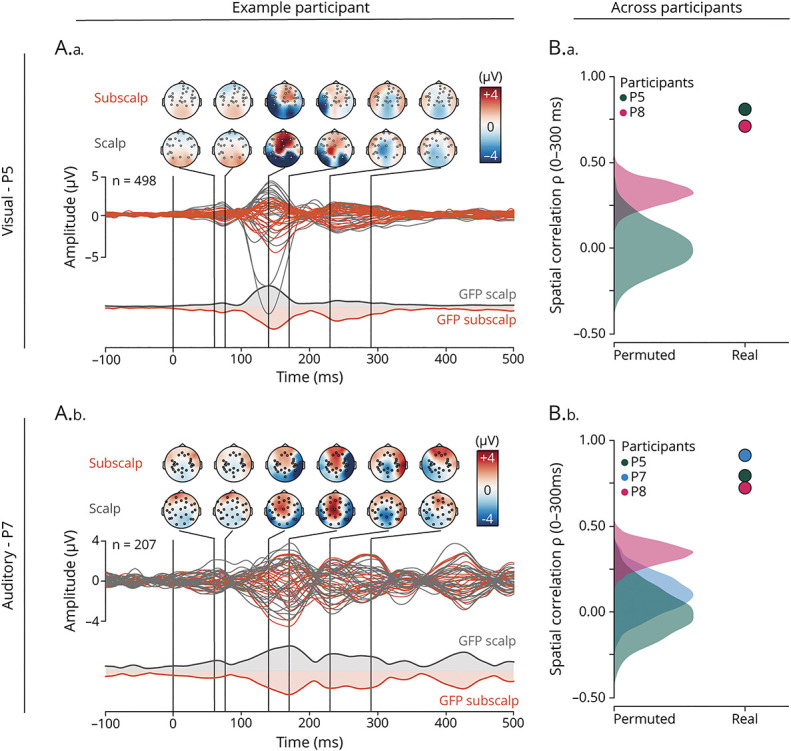
Evoked Potentials in the Subscalp and Scalp EEG Recordings (A.a., A.b.) Example of average visual (A.a., n = 498 trials) and auditory (A.b., n = 207 trials) evoked potentials in participants P5 and P7, respectively. Topoplots enable the comparison of the spatial distribution of the response at different time points after stimulation onset for both the subscalp (top) and scalp EEG (bottom). The shared colorbar represents the amplitude in microvolts. The average response for each contact is displayed as a butterfly plot (subscalp in orange, scalp in gray). The corresponding global field potential is also depicted, with inverted polarity for subscalp EEG for visual rendering. (B.a., B.b.) Individual spatial correlation between subscalp and scalp EEG recordings of visual and auditory evoked potentials in 2 (P5, P8) and 3 (P5, P7, and P8) participants, respectively. The mean spatial correlation coefficient (ρ) across the time range of 0–300 milliseconds is represented by dots, and the permutation distribution for each individual is shown as density plots. True spatial correlation (dots) was in each case above the null-distribution drawn from shuffled electrode labels.

Subscalp and scalp electrodes measured the potentials evoked by repetitive visual (flickering checkerboard) or auditory (short-lived pure tones) stimuli with a borderline (visual ICC [95% CI] = 0.26 [−0.04 to 0.52]) and strong agreement (auditory ICC [95% CI] = 0.80 [0.69–0.87]) and with a bias within the preset margin of noninferiority (bias [95% CI] = −0.63 μV [−1.05 to −0.12] ≥−1 μV for visual, −0.18 μV [−0.33 to −0.02] ≥−1 μV for auditory, eFigure 4, eTable 4). The topographic distribution of the evoked potentials was similar with a median (range) spatial correlation of 0.76 (0.72–0.80) (*p* < 0.05, surrogate-tested) for visual and 0.75 (0.70–0.85) (*p* < 0.05, surrogate-tested) for auditory stimulation (Figure 3).

### Sleep EEG Recordings

#### Sleep Oscillations

**Figure 4:**
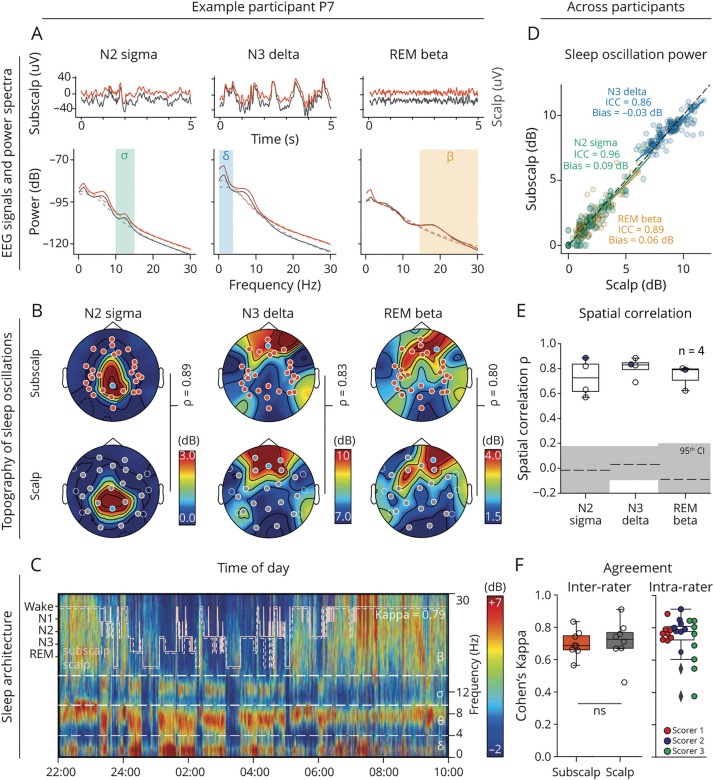
Subscalp and Scalp EEG Recordings During Sleep (A, top) Example of subscalp and scalp EEG signals (5 seconds excerpt) in 1 participant (P7) across non-rapid eye movement sleep (N2, N3) and REM sleep. (Bottom) Corresponding multitaper power spectral density showing oscillation characteristic of each sleep stage over 1 entire night of sleep (shown in C). (B) Corresponding topoplots showing peak power distribution for each characteristic sleep oscillation in subscalp (top) and scalp (bottom) signals for 1 participant (P7). (C) Corresponding multitaper spectrogram (1/f fit removed) for 1 night of sleep (subscalp contact EP3) along with scored hypnograms once based on subscalp (orange) and once on scalp EEG recordings (dashed white). (D) Scatterplot of peak power in 3 sleep oscillations (N2 sigma in green, N3 delta in blue, REM beta in yellow) measured with subscalp vs scalp EEG over 1 night of sleep in each participant (n = 8). Dots correspond to pairs of neighboring subscalp and scalp contacts (n = 161) color-coded by the measured sleep oscillation. The corresponding linear fits (full colored lines) are visually compared with the identity line (dashed diagonal). Corresponding ICCs are shown, and Bland-Altman bias calculations are in eFigure 4. (E) Boxplots showing the median (IQR) spatial correlation between subscalp and scalp EEG for characteristic sleep oscillations in each participant implanted with 4 tridents (dots, n = 4). Black dot is the example in A–C. Black dashed lines and gray shadow indicate the median and 95th percentile of surrogate data. (F) Left: Comparison of median (IQR) inter-rater reliability quantified as Cohen's κ among 3 sleep scorers using subscalp (orange) or scalp (gray) EEG signals to score 8 nights separately. Right: The median (IQR) intrarater agreement quantified as Cohen's κ among 8 nights scored separately with subscalp or scalp EEG signals by each of the 3 sleep scorers (color dots). ICC = interclass correlation coefficient; IQR = interquartile range.

The 2 recording modalities revealed the characteristic sleep oscillations at the expected topographical location in association with each of the main sleep states: central sigma oscillations (spindles) in non-REM sleep stage 2 (N2), frontal delta oscillations (slow waves) in the non-REM sleep stage 3 (N3), and frontal beta oscillations during REM sleep (Figure 4, A and B). Across participants (n = 8), subscalp was not inferior to scalp EEG in its ability to measure peak power in these specific sleep oscillations with a strong correlation and a lack of bias for N2 sigma power (ICC = 0.96 [0.94–0.97], bias = 0.09 [0.01–0.16] >−0.5 dB), for N3 delta power (ICC = 0.86 [0.82–0.90], bias = −0.03 dB [−0.13 to 0.07] >−0.5 dB) and for REM beta power (ICC = 0.89 [0.085–0.92], bias = 0.06 dB [−0.04 to 0.15] >−0.5 dB, Figure 4D, eFigure 4, eTable 4). Furthermore, topoplots drawn from subscalp and scalp EEG in 4 participants had moderate to strong spatial correlations for each sleep stage and characteristic oscillation: median (range) ρ = 0.73 (0.62–0.84) for N2 sigma power, ρ = 0.83 (0.79–0.85) for N3 delta power, and ρ = 0.79 (0.71–0.80) for REM beta power, all above values from surrogate data (Figure 4, B and E, n = 4).

#### Sleep Score Agreement

The current gold standard for determining sleep architecture is visual scoring (Figure 4C).^17^ We assessed the agreement among 3 trained scorers (E.v.M., C.F.-M., K.W.), who scored the same night for each participant (n = 8) generating one hypnogram based on subscalp EEG and another hypnogram based on scalp EEG (traces in Figure 4C). Using Cohen κ score, we found a similarly strong (*p* = 0.38, sign test) *inter*-rater agreement between hypnograms based on subscalp (median κ = 0.67 [0.66–0.75]) or scalp (median κ = 0.73 [0.67–0.76], Figure 4F) EEG, indicating that scorers agreed regardless of the recording modality. In addition, we found a similarly strong *intra*-rater agreement (median κ = 0.78 [0.72–0.81]) between hypnograms scored with the 2 modalities for the same night (scorer 1: 0.76 [0.74–0.79], scorer 2: 0.79 [0.79–0.83], scorer 3: 0.73 [0.59–0.81], Figure 4F), confirming that both modalities captured an equivalent sleep architecture.

### Ictal EEG Recordings

**Figure 5:**
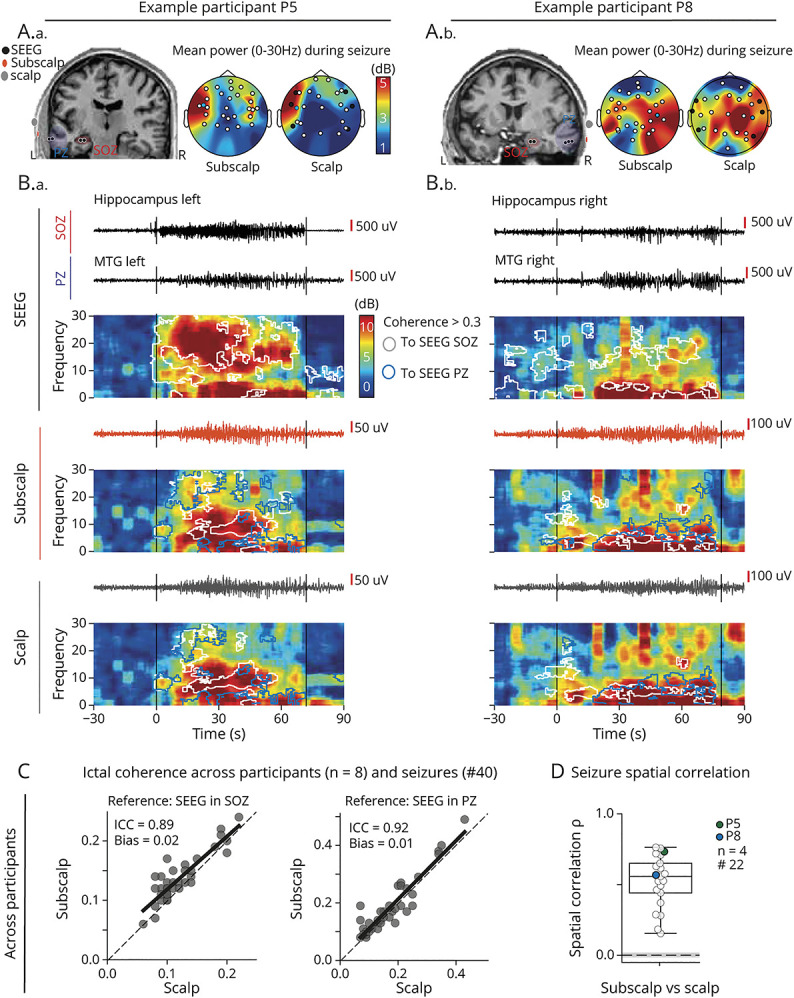
Epileptic Seizures Recorded With Intracranial, Subscalp, and Scalp EEG (A) For 2 participants (left P5, right P8), stereo-EEG contacts (black) are shown in an omniplanar slice of the MRI that captures the SOZ (red, hippocampus left/right), the PZ (blue, laterotemporal neocortex left/right), and neighboring subscalp (orange) and scalp (gray) electrode contacts. Accompanying topoplots show ictal activity visualized as the mean power increase during 1 seizure across frequencies 0.5–30 HZ. (B) For the same seizure, EEG signals from the stereo-EEG electrodes in the SOZ and PZ as well as neighboring subscalp and scalp electrodes are shown with the corresponding spectrogram (1/f fit removed). Segments of high spectral coherence (>0.3) with the SOZ or the PZ stereo-EEG are shown as white and blue contours, respectively. Note how extracranial EEG signals are coherent with SOZ and PZ signals at different frequencies, as the seizure propagates. (C) Scatterplot of ictal coherence of extracranial signals with ground-truth intracranial signals (left: from the SOZ, right: from the PZ), measured with subscalp vs scalp EEG in a variable number of seizures across participants (n = 8). Dots correspond to 1 pair of neighboring subscalp and scalp contacts for each seizure (n = 40). The linear fit (black line) is visually compared with the identity line (dotted diagonal). Corresponding ICCs are shown, and Bland-Altman bias calculations are in eFigure 4. (D) For 22 seizures among 4 participants implanted with 4 tridents, the time-average spatial correlation of spectral power increase across seizures. Dashed horizontal line and gray shading correspond to the median and 5th and 95th percentiles of surrogate data. ICC = interclass correlation coefficient; IQR = interquartile range; PZ = propagation zone; SOZ = seizure onset zone.

Of 51 seizures recorded with intracranial EEG (ground-truth) across 8 participants, we visually identified 40 (78%) in subscalp and 39 (76%) in scalp EEG (eTable 5). Twelve additional seizures (22% of all recorded seizures) were restricted to deep-seated cortical areas thus not visible with extracranial (subscalp or scalp) EEG recordings and therefore excluded from subsequent analyses. Leveraging the availability of a ground-truth intracranial EEG in these participants, we analyzed individual seizures in terms of their SOZ and PZ, respectively, the latter often being better recorded extracranially. In topographical examples, we found that both subscalp and scalp EEG had similar localization ability (at the lobar level) in left-temporal (P5, Figure 5 A.a.) or right-temporal seizures (P8, Figure 5 A.b.). In individual seizures, recorded extracranial signals could be coherent with the SOZ, the PZ, or both (Figure 5B). Across participants (n = 8), subscalp was noninferior to scalp EEG in its ability to measure spectral coherence (C, average over 0.5–30 Hz) with ground-truth stereo-EEG in the SOZ and PZ. Indeed, the correlation between the subscalp and scalp EEG was strong and bias absent for their measured coherence with the SOZ (ICC = 0.90 [0.82–0.95], bias = 0.02 [0.01–0.02] >−0.03) and the PZ (ICC = 0.92 [0.86–0.96], bias = 0.01 [0.00–0.02] >−0.03, Figure 5C, eFigure 4, eTable 4). Spatial correlation in ictal activity maps between subscalp and scalp EEG (averages of spectral power increase over the entire seizure) was moderate (ρ = 0.56 [0.44–0.65]) and significant (*p* < 0.05, surrogate-tested, Figure 5D, n = 4).

### Interictal EEG Recordings

**Figure 6:**
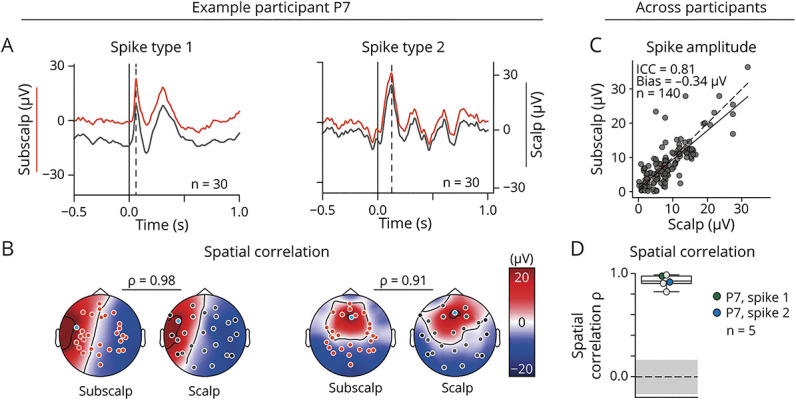
Interictal Epileptic Spikes Recorded With Subscalp and Scalp EEG (A) Example of mean EEG signal (n = 30 spikes) from 1 subscalp (orange) and 1 scalp (gray) contact for 2 different spike types (left and right) identified in 1 participant (P7). Vertical full and dashed lines show times of spike onset and peak amplitude, respectively. (B) Corresponding subscalp (left) and scalp (right) topographic distributions of mean spike peak amplitudes across contacts for the 2 spike types. Inferior temporal scalp contacts are shown as black dots. (C) Scatterplot of mean interictal spike peak amplitude measured with subscalp vs scalp EEG in participants with full-head coverage (n = 4, P7 is represented twice for the 2 spike types). Dots correspond to pairs of neighboring subscalp and scalp contacts. The linear fit is shown in black and visually compared with the identity line (dotted diagonal). The corresponding ICC is shown, and Bland-Altman bias calculations are in eFigure 4. (D) Spatial correlation of all topographic distribution of 5 identified spike types in 4 participants. The expected CI's median (line) and 95th percentile (gray shadow) based on surrogate testing is shown. ICC = interclass correlation coefficient.

Across 4 participants (n = 4), subscalp was noninferior to scalp EEG in its ability to measure and localize average spike peak-amplitude from 30 peak-aligned interictal epileptic spikes. Correlation between these measurements was strong (ICC = 0.81 [0.74–0.87]) and bias absent (bias = −0.34 μV [−0.98 to 0.3] >−1 μV, Figure 6, A and B, eFigure 4, eTable 4). The corresponding topographic maps showed a strong spatial correlation (ρ = 0.92 [0.91–0.98], *p* < 0.05, surrogate-tested, Figure 6C), suggesting a similar ability to localize the dipoles generating such discharges. In 1 participant (P7), spikes were bifocal with different fields.

## Discussion

This single-arm interventional clinical trial in 8 adults with focal epilepsy met our predefined primary end point: It demonstrated the feasibility of inserting a novel electrode device (Epios) beneath the skin to record subscalp EEG from the entire head for up to 9 days. The lack of device-related serious adverse events attests to the trial participants' overall safety. The performance of the device was noninferior to conventional 10-20 scalp EEG in measuring physiologic brain oscillations and pathologic discharges, and full-head subscalp EEG showed efficacy in localizing these brain signals of interest.

The main limitation of this trial is its short duration (up to 9 days) during the hospital stay of a limited number of participants. Eight of 14 screened patients (57%) agreed to participate in this study, which offered no direct clinical benefit, but potential risks. By leveraging the opportunity to perform an interventional trial in participants undergoing intracranial EEG monitoring for clinical reasons, we acknowledge barriers to participation and a selection bias toward more severe epilepsies and/or deep-seated seizure foci. However, ground-truth intracranial EEG was advantageous for some of the uniquely informative analyses provided here. Although percutaneous subscalp EEG monitoring may be useful in the hospital (e.g., in critically ill patients),^24^ we expect a higher interest from the broader population with pharmacoresistant epilepsy in using a fully implanted subscalp EEG system in daily life.^13-15^ This will require connecting the tested electrode lead to an implanted transceiver for transcutaneous data collection, which is feasible^14,15^ and will be the focus of a future clinical trial in ambulatory patients.

We showed initial evidence for safety (primary outcome, Table 1 and Figure 1), supporting the opinion that subscalp devices are generally safe,^25^ although the current scarcity of available data overall forbids any premature conclusion.^11,12^ Our subscalp trident electrodes can be inserted over the entire head through 4 small incisions ≤1 cm in a 1-hour minimally invasive procedure, effectively translocating the conventional EEG coverage to the subscalp space. We found 5 minor (none serious) procedure-related adverse events that were foreseen and self-limited. Postoperative pain was tolerable, and palpation of the scalp above the inserted electrodes was painless. Thus, we found no definitive surgical concern that should limit head coverage for acquiring subscalp EEG.

We also showed evidence for device performance and diagnostic efficacy (secondary outcome), in that our system provided meaningful physiologic (Figures 2–4) and pathologic signals (Figures 5 and 6) for evaluation by an expert viewer or quantitative analyses. Visually, spectrally, and topographically, the signals recorded from subscalp EEG were more similar (and noninferior) to scalp EEG than intracranial EEG.^26^ They contained well-characterized, region-specific electrographic signatures of the sleep-wake cycle^27^ that expert viewers could reliably use to infer sleep stages. Furthermore, subscalp EEG could localize interictal and ictal epileptiform discharges from different foci, similarly to scalp EEG. We recorded mesiotemporal, laterotemporal, and frontoparietal seizures across patients and localized different spike sources across and within patients.

Previously, a few implantable systems have been able to record limited EEG data in ambulatory patients with epilepsy. Existing intracranial devices for palliative neurostimulation (RNS System^9^ and Medtronic DBS^10^) have limited capability for recording raw EEG and/or require knowledge of the seizure focus for electrode placement. Two existing subscalp systems (UNEEG^15^ and Minder^14^) and 2 in development (Neuroview^28^ and Braincare) all severely limit head coverage to 1 single electrode lead track restricting their indication to special cases.^29^ Crucially, the novel subscalp electrode device tested here can overcome these limitations because it obviates the need to know seizure localization a priori. Indeed, full-head coverage—our main differentiating strength—finds its greatest importance in the ability to detect and localize sources of recorded physiologic or pathologic signals with lobar or sublobar precision.

Thus, full-head subscalp EEG has promise for ultra-long-term recordings and is noninferior to the standard of care (scalp EEG), including known disadvantages (electromyogram contamination, low spatial resolution) and advantages (high spatial coverage) over current intracranial and subscalp EEG devices. In pharmacoresistant epilepsy, specifically, full-head coverage is necessary to characterize single or multiple epileptic foci. Over months, the device could enable increasingly refined localization of seizures, while guiding medical treatment optimization.^1^ Shall this approach fall short, the collected data would help with presurgical planning,^1^ the currently recommended next step. A better stratification of candidates to epilepsy surgery and selection of implantation targets (intracranial EEG) may help avoid surgical failure resulting from the variability of seizure foci across, and sometimes within patients.

This study represents a proof of concept toward an implantable full-head subscalp EEG system for ultra-long-term monitoring of brain oscillations and discharges. Among implantable technologies for brain disorders, our minimally invasive system is positioned for earlier use along a patient's journey. Neurology as a field would benefit from a portable device that can reliably confirm the nature of rare or frequent neurologic events and monitor as well as localize pathologic brain activity for treatment guidance and optimization. With ever-growing data processing capability, our innovation will likely contribute to the emergence of a new era in ambulatory brain monitoring, seizure forecasting, and disease management for epilepsy as well as other brain disorders.

## Appendix Authors

**Table:**

Name	Location	Contribution
Ellen van Maren, PhD	NeuroTec, Center for Sleep-Wake-Epilepsy, Center for Experimental Neurology, Department of Neurology, Inselspital Bern, University Hospital, University of Bern, Switzerland	Drafting/revision of the manuscript for content, including medical writing for content; analysis or interpretation of data
Sigurd L. Alnes, PhD	NeuroTec, Center for Sleep-Wake-Epilepsy, Center for Experimental Neurology, Department of Neurology, Inselspital Bern, University Hospital, and Institute of Computer Science, University of Bern, Switzerland	Drafting/revision of the manuscript for content, including medical writing for content; analysis or interpretation of data
Janir Ramos da Cruz, PhD	Wyss Center for Bio and Neuroengineering, Geneva, Switzerland	Drafting/revision of the manuscript for content, including medical writing for content; analysis or interpretation of data
Aleksander Sobolewski, PhD	Wyss Center for Bio and Neuroengineering, Geneva, Switzerland	Drafting/revision of the manuscript for content, including medical writing for content; analysis or interpretation of data
Cecilia Friedrichs-Maeder, MD	NeuroTec, Center for Sleep-Wake-Epilepsy, Center for Experimental Neurology, Department of Neurology, Inselspital Bern, University Hospital, University of Bern, Switzerland	Drafting/revision of the manuscript for content, including medical writing for content; analysis or interpretation of data
Katharina Wohler, MD	NeuroTec, Center for Sleep-Wake-Epilepsy, Center for Experimental Neurology, Department of Neurology, Inselspital Bern, University Hospital, University of Bern, Switzerland	Drafting/revision of the manuscript for content, including medical writing for content; analysis or interpretation of data
Sabry L. Barlatey, MD, PhD	Department of Neurosurgery, Inselspital Bern, University Hospital, University of Bern, Switzerland	Drafting/revision of the manuscript for content, including medical writing for content; major role in the acquisition of data
Sandy Feruglio	NeuroTec, Center for Sleep-Wake-Epilepsy, Center for Experimental Neurology, Department of Neurology, Inselspital Bern, University Hospital, University of Bern, Switzerland	Major role in the acquisition of data
Markus Fuchs	NeuroTec, Center for Sleep-Wake-Epilepsy, Center for Experimental Neurology, Department of Neurology, Inselspital Bern, University Hospital, University of Bern, Switzerland	Major role in the acquisition of data
Ioannis Vlachos, PhD	Wyss Center for Bio and Neuroengineering, Geneva, Switzerland	Drafting/revision of the manuscript for content, including medical writing for content
Jonas Zimmermann, PhD	Wyss Center for Bio and Neuroengineering, Geneva, Switzerland	Drafting/revision of the manuscript for content, including medical writing for content
Tiago Bertolote, MSc	Wyss Center for Bio and Neuroengineering, Geneva, Switzerland	Drafting/revision of the manuscript for content, including medical writing for content
Werner J. Z'Graggen, MD	Department of Neurosurgery, Inselspital Bern, University Hospital, University of Bern, Switzerland	Drafting/revision of the manuscript for content, including medical writing for content; major role in the acquisition of data
Athina Tzovara, PhD	NeuroTec, Center for Sleep-Wake-Epilepsy, Center for Experimental Neurology, Department of Neurology, Inselspital Bern, University Hospital, and Institute of Computer Science, University of Bern, Switzerland	Drafting/revision of the manuscript for content, including medical writing for content; analysis or interpretation of data
John Donoghue, PhD	Department of Neuroscience, Brown University, Providence, RI	Drafting/revision of the manuscript for content, including medical writing for content; study concept or design
George Kouvas, MSc	Wyss Center for Bio and Neuroengineering, Geneva, Switzerland	Drafting/revision of the manuscript for content, including medical writing for content; study concept or design
Kaspar Schindler, MD, PhD	NeuroTec, Center for Sleep-Wake-Epilepsy, Center for Experimental Neurology, Department of Neurology, Inselspital Bern, University Hospital, University of Bern, Switzerland	Drafting/revision of the manuscript for content, including medical writing for content; major role in the acquisition of data; study concept or design
Claudio Pollo, MD	Department of Neurosurgery, Inselspital Bern, University Hospital, University of Bern, Switzerland	Drafting/revision of the manuscript for content, including medical writing for content; major role in the acquisition of data; study concept or design
Maxime O. Baud, MD, PhD	NeuroTec, Center for Sleep-Wake-Epilepsy, Center for Experimental Neurology, Department of Neurology, Inselspital Bern, University Hospital, University of Bern, Switzerland	Drafting/revision of the manuscript for content, including medical writing for content; major role in the acquisition of data; study concept or design; analysis or interpretation of data

## Glossary

ICC: interclass correlation coefficient
IQR: interquartile range
PZ: propagation zone
SOZ: seizure onset zone
